# Metabolically Healthy Obesity—Heterogeneity in Definitions and Unconventional Factors

**DOI:** 10.3390/metabo10020048

**Published:** 2020-01-27

**Authors:** Inês Brandão, Maria João Martins, Rosário Monteiro

**Affiliations:** 1 Departament of Biomedicine, Biochemistry Unit, Faculty of Medicine, University of Porto, Al. Prof. Hernâni Monteiro, 4200-319 Porto, Portugal; inesm.brandao@gmail.com (I.B.); mmartins@med.up.pt (M.J.M.); 2 i3S—Instituto de Investigação e Inovação em Saúde, University of Porto, 4200-135 Porto, Portugal; 3 Centro de Apoio Tecnológico Agro Alimentar (CATAA), Zona Industrial de Castelo Branco, 6000-459 Castelo Branco, Portugal; 4 Administração Regional de Saúde-Norte, Unidade de Saúde Familiar Pedras Rubras, Agrupamento de Centros de Saúde Maia-Valongo, 4470-105 Maia, Portugal

**Keywords:** adipocyte hypertrophy, metabolic inflammation, obesity phenotypes, metabolically healthy obese phenotype, metabolically unhealthy normal-weight phenotype, metabolically unhealthy obese phenotype, persistent organic pollutants, gut microbiota, inflammasome, endocannabinoid system

## Abstract

The concept of heterogeneity among obese individuals in their risk for developing metabolic dysfunction and associated complications has been recognized for decades. At the origin of the heterogeneity idea is the acknowledgement that individuals with central obesity are more prone to developing type 2 diabetes and cardiovascular disease than those with peripheral obesity. There have been attempts to categorize subjects according to their metabolic health and degree of obesity giving rise to different obese and non-obese phenotypes that include metabolically unhealthy normal-weight (MUHNW), metabolically healthy obese (MHO), and metabolically unhealthy obese (MUO). Individuals belonging to the MHO phenotype are obese according to their body mass index although exhibiting fewer or none metabolic anomalies such as type 2 diabetes, dyslipidemia, hypertension, and/or unfavorable inflammatory and fribinolytic profiles. However, some authors claim that MHO is only transient in nature. Additionally, the phenotype categorization is controversial as it lacks standardized definitions possibly blurring the distinction between obesity phenotypes and confounding the associations with health outcomes. To add to the discussion, the factors underlying the origin or protection from metabolic deterioration and cardiometabolic risk for these subclasses are being intensely investigated and several hypotheses have been put forward. In the present review, we compare the different definitions of obesity phenotypes and present several possible factors underlying them (adipose tissue distribution and cellularity, contaminant accumulation on the adipose tissue, dysbiosis and metabolic endotoxemia imposing on to the endocannabinoid tone and inflammasome, and nutrient intake and dietary patterns) having inflammatory activation at the center.

## 1. Introduction

Obesity, defined by the World Health Organization (WHO) as abnormal or excessive fat accumulation that may impair health, constitutes a rising threat to public health and welfare [1] and has become an epidemic in both developed and developing countries. Obesity is not only intimately associated with the prevalence of chronic low-grade inflammation, underlying systemic metabolic dysfunction, but also linked to an elevated risk of premature death as a result of type 2 diabetes (T2DM), non-alcoholic fatty liver disease (NAFLD), cardiovascular diseases (CVD), and certain types of cancer [2,3,4,5,6,7,8,9,10,11].

In recent years, the existence of heterogeneity among obese subjects in what regards their cardiometabolic risk has come to light, with a subgroup of these individuals being reported to be more resilient to metabolic, inflammatory, and/or fibrinolytic dysfunctions and its associated complications [3,6,7,9,12,13,14,15,16,17]. Remarkably, a subset of non-obese subjects, on the other hand, may present metabolic, inflammatory, and/or fibrinolytic abnormalities commonly found in the obese [18]. Definitions of different obesity and normal-weight phenotypes have emerged and will be discussed here. Although they are still a matter of debate, these definitions have practical consequences. In addition to high health care costs, obesity and cardiometabolic dysfunction also lead to economic and social burden explained by lost work days, lower productivity at work, and higher disability and mortality [19]. Therefore, it becomes pivotal to allocate rational resources tailored for the prevention and treatment of according to the health phenotype [7,11,16,20,21]. Obesity and cardiometabolic dysfunction are diseases with a very complex management due to their multifactorial nature involving environmental, genetic, and psychosocial factors interacting through intricate networks [22].

Obesity closely associates with a chronic low-grade inflammation, triggered by metabolic surplus [23]. Expansion of adipose tissue (AT) is considered a central element in obesity-related inflammation, with other organs and tissues being pivotal sources of inflammatory mediators during obesity. For instance liver, pancreas, and muscle can simultaneously contribute to the inflammatory milieu and be affected by it [24,25]. Adipose-originated metabolic inflammation has a close relationship with insulin resistance, being a determinant player for metabolic syndrome development, and has been under investigation for decades [25]. Inflammation and insulin resistance have been considered by many the main instigators of metabolic syndrome [26,27], being hallmarks of AT dysfunction. The list of triggers for AT inflammation is continuously growing and extends far beyond positive energy balance. This inflammatory signaling centered on the AT, with many possible culprits, leads to a vicious cycle that perpetuates AT dysfunction, hinders metabolic adaptation [28,29], and may be at the basis of unhealthy, opposed to heathy, obesity.

## 2. The Diverse Weight-Metabolic Phenotypes

Despite the presence of cardiometabolic disturbances in a vast majority of obese individuals, a considerable amount of people with a high body mass index (BMI) and excessive AT lack most of the metabolic, inflammatory, or fibrinolytic abnormalities and comorbidities typically associated with obesity although experiencing the same mechanical complications observed in other obese subjects [3,6,7,9,12,14,15,30,31,32]. Compared with age- and BMI-matched metabolically unhealthy counterparts, the metabolically healthy subjects have a lower risk of cardiovascular and liver diseases, may present normal insulin sensitivity and glucose metabolism regardless of the excessive amount of body fat [33]. On the other hand, a subset of normal-weight individuals who are metabolically unhealthy was identified in the 1980s by Ruderman et al., who suggested to characterize them according to certain parameters such as hyperinsulinemia, insulin resistance, hypertriglyceridemia, and coronary heart disease [18]. More recently, postmenopausal women who are metabolically unhealthy were associated with increased risk for breast cancer regardless of normal BMI, when compared to healthy counterparts [34]. Obese and non-obese phenotypes that include metabolically unhealthy normal-weight (MUHNW), metabolically healthy obese (MHO), and metabolically unhealthy obese (MUO) have been widely reported within clinical and epidemiological studies (see below). In MUHNW and MHO individuals, obesity is not linked to its usual metabolic, inflammatory, and/or fibrinolytic consequences, offering insight into risk factors that are largely independent of overall obesity (MUHNW) or risk factors associated with obesity that are largely independent of adiposity-induced abnormalities (MHO) [35]. Still, the phenotype categorization remains a matter of debate as it lacks standardized definitions (discussed below).

### 2.1. The Metabolically Unhealthy Normal-Weight Phenotype

The MUHNW phenotype is characterized by a normal BMI (18.5–25 kg/m^2^) associated with reduced lean body mass and increased levels of adiposity and ectopic fat distribution, with augmented visceral AT (vAT) and abdominal subcutaneous AT (sAT) which is a distinctive feature of this category [36,37]. MUHNW individuals tend to neglect prevention or clinical treatment, thus presenting an increased prevalence of clinical aspects often found in metabolic syndrome, such as low insulin sensitivity, increased blood pressure, decreased high density lipoprotein (HDL)-cholesterol, and high levels of triglycerides and systemic inflammatory markers [38,39,40]. In addition to the risk of developing metabolic syndrome, the MUHNW phenotype is characterized by increased risk of T2DM and CVD. Moreover, elderly people presenting this phenotype show a higher risk of CVD-related and all-cause mortality [41]. A standardized definition of MUHNW is still lacking, thus compromising an early diagnosis of this condition necessary for a suitable risk management [42] and preventive measures. During a prospective cohort study, Lee et al. proposed a triglyceride-glucose index (ln (fasting triglycerides [mg/dL] × fasting plasma glucose [mg/dL])/2) to be used as simple diagnostic criterion for MUHNW (above 8.82 for men and 8.73 for women) among normal weight people [41].

### 2.2. The Metabolically Healthy Obese Phenotype

Andres and Sims pioneered the proposal of the existence of MHO subjects, although without completely characterizing this clinical entity [12,43,44]. Most articles suggest that individuals belonging to the MHO phenotype are obese with BMI over 30 kg/m^2^ not exhibiting metabolic anomalies such as T2DM, dyslipidemia, hypertension, and/or unfavorable inflammatory and fibrinolytic profiles [3,7,11,12]. The absence of a unique widely universal definition and the inconsistencies in the criteria used between studies for the MHO concept explain the variability found in the prevalence of MHO phenotype, ranging from 6% to 75% of the obese population, as summarized by Rey-López et al. [16,45]. Noteworthy, Kuk et al. proposed MHO is a rare phenotype, suggesting it accounts for only 1.3% of the U.S. population [46].

Several underlying factors have been proposed to explain the healthier profile in individuals with MHO, among which are lower vAT and ectopic fat accumulation (including decreased hepatic steatosis) when compared to the more expandable subcutaneous fat depots [13]. A low inflammatory degree and low immune cell infiltration into AT is also a feature of MHO, as further discussed ahead [8,47].

Although MHO subjects present preserved high insulin sensitivity, a favorable lipid profile (e.g., reduced levels of triglycerides), increased levels of adiponectin and/or absence of hypertension and, so, a reduced risk of developing T2DM and CVD, there is no evidence protection is permanent [5,35,36,37,42,48,49]. For instance, Marini et al. showed that a group of 20 MHO women presented a less positive metabolic profile compared with a control group of 80 non-obese women [40]. These MHO women had significantly increased blood pressure and carotid artery intima-media thickness and lower concentrations of HDL-cholesterol, which could represent early signs of atherosclerosis [40]. Oflaz et al. further revealed that 24 MHO women, although showing a normal metabolic profile, presented signs of deteriorated endothelial function and atherosclerotic changes when compared with their healthy lean counterparts (*n* = 14) [50]. Additionally, Meigs et al. reported that insulin-sensitive obese individuals present a three-times higher risk of T2DM (after 11 years of follow up of 2902 men and women) when compared to insulin-sensitive normal-weight subjects, pointing towards the diabetogenic nature of obesity even in the initial absence of insulin resistance [35]. In the same line, a study comprising 6011 men and women from the Third National Health and Nutrition Examination Survey (NHANES III), with public-access mortality data linkage, showed that, in the absence of metabolic abnormalities, obesity is associated with augmented all-cause mortality risk [46]. From the community-based Uppsala Longitudinal Study of Adult Men, comprising 1675–1758 participants, Arnlöv et al. observed that overweight or obese middle-aged men without metabolic syndrome were at increased risk for diabetes after 20 years of follow up [49]. After more than 30 years of follow up, the same subjects also presented increased risk for cardiovascular events and all-cause mortality [5]. More recently, Espinosa De Ycaza et al. conducted a retrospective cohort involving 1805 MHO and 3047 metabolically healthy normal-weight (MHNW) adults with a median follow-up of 15 years and concluded that MHO individuals are more likely to develop metabolic complications than MHNW, with this difference being accentuated when MHO gained weight [51].

In the following section we will discuss the multiple different definitions for MHO from several sources aiming to show that there is heterogeneity in them, possibly blurring the distinction between obesity phenotypes and confounding the associations with health outcomes. Stratification of obese individuals taking into consideration their metabolic health phenotype is relevant for the establishment of the most suitable (even personalized) lifestyle, therapeutic and/or surgery strategies [7,11,20,52,53].

#### The Multiple Definitions of Metabolically Healthy Obesity

There are more than a few distinct definitions of the concept of MHO (also named as benign or uncomplicated obesity), which identify subpopulations of obese individuals presenting different levels, in a more favorable profile, of metabolic, inflammatory, and fibrinolytic activity as well as immune and liver function abnormalities. Some authors argue that the distinct MHO definitions support the concept that (a) MHO does not describe a biologically fixed, defined, or stable phenotype and (b) MHO represents the extreme healthier phenotype of continuous associations between increased obesity and several diverse metabolic, inflammatory, and/or fibrinolytic activity dysfunctions along with immune and/or liver function impairments. Some authors claim that MHO provides short- but not long-term protection against obesity-associated comorbidities, what supports its transient nature. Different definitions of MHO identify different subgroups of obese individuals what is suggestive of distinctive etiologies for this phenotype. Despite a multitude of studies focusing on MHO and its characteristics, a large disparity both in the criteria used to define this phenotype and in the health outcomes analyzed still exists, hence leading to a difficult comparison among published data [6,7,11,16,20,30,31,32,42,52,53,54,55,56,57,58,59,60,61,62,63,64,65,66,67,68]. Additionally, variables that include lifestyle (diet (food groups and macro- and micronutrients intakes), smoking status, alcohol consumption, physical activity (for example motor activity, sedentary behavior, and exercise)), physical fitness (cardiorespiratory fitness, functional capacity, muscular strength, etc.), psychosocial stress, ethnicity, gender, pubertal development, or age account for the complexity in evaluating MHO although they are not included in current MHO definitions [6,7,9,11,16,55,56,57,60,62,63,64,67,69,70].

MHO definitions differently include the parameters that are presented in Table 1. Additionally, distinct cut-off values (sometimes not based on an established value but instead dependent on the population being studied) and number of inclusion criteria considered for the setting of the MHO phenotype are used [3,5,6,7,9,10,11,12,15,16,20,30,31,32,35,38,40,42,46,48,52,53,54,55,56,57,58,59,61,62,63,64,65,68,69,70,71,72,73,74,75,76,77,78,79,80,81,82,83,84,85,86,87,88,89].

In order to define MHO, the aforementioned parameters have been organized bearing in mind the following [16,30,31,32,35,42,53,56,59,66]: (1) metabolic syndrome criteria; (2) a combination of metabolic syndrome criteria and insulin sensitivity; (3) a combination of metabolic syndrome criteria and inflammatory markers (C-reactive protein) (Table 2, [52,61,75,80]); (4) a combination of metabolic syndrome score, insulin sensibility/homeostasis, and inflammatory markers (C-reactive protein, fibrinogen and/or white blood cell (WBC) count) (Table 2, [12,54,59,77,86]); and (5) insulin sensitivity/glucose homeostasis. The MHO definition most frequently used relies on four metabolic syndrome components: HDL-cholesterol, triglycerides, and glucose levels in addition to blood pressure values [16]. With or without some modification or expansion, the metabolic syndrome definition included in the MHO classification is based on the guidelines by the International Diabetes Federation, National Cholesterol Education Program Expert Panel and the American Heart Association/National Heart, Lung and Blood Institutes (in 2005) as well as the joint guidelines of major International Organizations (in 2009) [7,9,10,12,16,30,31,32,35,48,52,56,61,63,65,75,76,79,81,82,84,86,88,89,90,91,92,93,94,95,96,97,98,99,100,101,102] (some examples of their use were included in the references).

The relevance of MHO definition harmonization has been discussed and the aim of obtaining a unique MHO definition has been disclosed [11,16,32,53,55,56,57,59]. Interestingly, a combination of obesity and absence of components of the metabolic syndrome (in some definitions with the exception of waist circumference) has been suggested as a potential MHO definition and is already in use [55,56,57,58,64]. Table 2 presents some examples of MHO definitions that include inflammatory parameters.

### 2.3. The Metabolically Unhealthy Obese Phenotype

At last, the MUO phenotype is defined by a BMI over 30 kg/m^2^ and body fat percentage over 30% [103,104,105]. MUO patients typically reveal an ectopic fat distribution with excessive accumulation of vAT and are considered at risk to develop major health problems (including metabolic syndrome progression, T2DM, and CVD) and, consequently, present higher mortality [33,105,106]. Given the dissimilarities observed in metabolic profiles among people within the same categories of BMI, several authors considered that instead of the amount of AT other factors such as composition and distribution of body fat are determinants of metabolic function [107,108,109].

## 3. Metabolic Inflammation

Nowadays, although it is well-established that inflammation is a major contributor for the development of obesity-related metabolic disorders (e.g., insulin resistance and T2DM), the underlying molecular mechanisms leading to the inflammatory state in AT remain only partially understood [22,110].

The first report establishing a link between inflammation and obesity revealed augmented levels of tumor necrosis factor α (TNFα) in AT of obese mice compared with lean controls [111]. Since then, a wealth of studies focusing on inflammation in obesity revealed a growing list of proinflammatory cytokines produced by adipocytes and infiltrating macrophages that are increased in obese when compared to lean subjects, such as interleukin (IL) 6, IL1β, IL18, leptin, CC-chemokine ligand 2 (CCL2), and resistin [110,112,113].

In obesity, inflammatory responses present a chronic low-grade condition, distinct from the features observed in classical inflammation elicited by infection, cancer, or injury [114]. The terms “meta-inflammation”, signifying metabolically-triggered inflammation [114], or “para-inflammation” for an intermediate state between basal and full inflammatory states [115], were proposed to name the inflammatory state observed in obesity. Both metabolic and immune systems are highly interdependent, with common cellular machinery and sharing modulators and regulators, that include hormones, cytokines, signaling protein mediators, transcription factors, and bioactive lipids [24,116].

Besides functioning as energy reservoir, thermal regulator, and mechanical protector for internal organs, AT is a central metabolically active endocrine organ. This tissue plays a role in energy homeostasis and insulin sensitivity through secretion of a wide range of molecules, such as adipokines, cytokines, hormones, and growth factors [117,118].

Inflammatory processes in the AT are now regarded as contributors to obesity-related metabolic disorders [24,119]. The energy surplus in AT has been shown not only to induce proinflammatory responses but also to lead to endoplasmic reticulum stress, hypoxia, mitochondrial defects, and ultimately, to systemic insulin resistance [119,120,121]. Augmenting lipid and carbohydrate substrates results in a higher demand on the mitochondrial electron transport chain. The increased demand for nutrient oxidation along with the augmented hypoxia, due to insufficient AT vascularization, generate abnormally high amounts of reactive oxygen species [22,122]. Oxidative stress leads to activation of major inflammatory kinases, such as c-Jun N-terminal kinase (JNK), p38 mitogen-activated protein kinases (MAPK), and inhibitor of kappa B kinase (IKK), that may directly interfere with insulin signaling, or indirectly via induction of the nuclear factor kappa-light-chain-enhancer of activated B cells (NFκB), and increase of proinflammatory cytokine and chemokine production [22,123].

Understanding fully how metabolic homeostasis deteriorates and relates to inflammation and pinpointing triggering factors for inflammation in obesity still poses an extremely hard challenge.

### Adipose Tissue Inflammation and Pathogenesis of Obesity

#### Adipose Tissue Cellularity, Remodeling, and Inflammation

White AT (wAT) plays a key role in mediating the systemic inflammation observed in certain obesity phenotypes. Chronic nutrient overload results in excessive fat accumulation and implicates hyperplasia (increase in adipocyte number) and adipocyte hypertrophy (increase in cell size) [110,124]. Growing adipocyte size requires a constant remodeling in the extracellular matrix of the AT, that, if insufficient will lead to vascularization and innervation deficits [124,125].

Srdic et al. found that MUHNW subjects reveal higher adipocyte hypertrophy in vAT when compared to healthy counterparts [89,125,126]. In the same line, MHO subjects were associated with smaller adipocytes relative to MUO controls [89,125]. O’Connell et al. reported a significant increase of the mean omental adipocyte size in MUO when compared to MHO. Adipocyte size strongly correlates with metabolic parameters, such as insulin resistance, triglyceride levels, hepatic steatosis, and fibrosis. A higher degree of steatosis was found in MUO (43%) than in MHO (3%). The size of adipocytes was suggested to be more relevant than the actual size of fat depot [127]. Later, O’Connell revealed AT in MHO individuals presented lower levels of preadipocyte factor-1 (Pref-1), a known inhibitor of preadipocyte differentiation, and a more favorable inflammatory profile, with lower numbers of macrophages, lower levels of TNFα, monocyte chemotactic protein-1 (MCP1), and granulocyte colony-stimulating factor, and higher levels of adiponectin [128]. Consistently, McLaughlin et al. reported that an expanded population of subcutaneous small adipose cells together with lower expression of preadipocyte differentiation markers (e.g., peroxisome proliferator-activated receptor γ (PPARγ) 1 and 2, glucose transporter type 4 (GLUT4), adiponectin) are linked to insulin resistance, thus suggesting impaired cell differentiation in this tissue may contribute to obesity-associated insulin resistance [74]. The importance of adipocyte size could potentially be explained by the overflow hypothesis that suggests adipocytes have a limit to store lipids. When this limit is reached, reminiscent fatty acids start to “overflow” to ectopic locations such as muscle, heart or liver, leading to cardiovascular and metabolic risk (e.g., hepatic insulin resistance) [127,129,130,131]. Overall, the capacity to recruit new or small adipocytes seems to be associated with a better metabolic health status [31,89,125,126,127,128].

#### Adipose Tissue Macrophages

Adipocyte hypertrophy, followed by augmented release of proinflammatory cytokines, promotes AT infiltration with immune cells and phenotypic changes in resident immune cells [110,125]. Weisberg et al. reported an increase of AT proinflammatory macrophages in obese individuals, accompanied by overexpression of TNFα, IL6, inducible nitric oxide synthase, transforming growth factor β1, and C-reactive protein (CRP), among others [121]. Additionally, Xu et al. showed an upregulation of multiple inflammation and macrophage-specific genes in wAT of genetic and high-fat-induced obesity mouse models. The observed upregulation preceded the increase in serum insulin levels. Furthermore, authors reported a significant infiltration of macrophages in histological samples of wAT in obese mice and proposed AT infiltrating macrophages and their inflammatory pathways to be major contributors to the pathogenesis of obesity-induced insulin resistance [120]. Diet-induced obesity has been shown to provoke a phenotypic switch in AT macrophage polarization into the proinflammatory M1-state, which relates to insulin resistance [132]. Interestingly, AT resident macrophages were reported to be higher in vAT when compared to sAT [133], which could relate with the worse metabolic risk profile observed in subjects with vAT accumulation [134]. Consistently, vAT adiposity was reported to have a central role in the development of insulin resistance and inflammation [125,135,136].

MHO has been linked to a low inflammatory degree, with reduced WBC counts and low levels of TNFα, IL6, and CRP identified in the plasma [8,47]. Additionally, a normal adipose function associated with lower immune cell infiltration into AT and a normal adipokine secretion pattern was reported in MHO individuals [137].

#### Patterns of Adipose Tissue Distribution

Vague firstly reported a distinct fat deposition between sexes, with a worse metabolic profile being related to the android (male) when compared to the gynoid (female) body type [138,139]. These two terms are used to classify obesity in terms of fat distribution. In the android fat distribution, individuals present accumulation of AT mainly around the trunk and upper body (e.g., abdomen, chest). This central obesity pattern often named “apple-shaped” is more commonly found in men. In the gynoid fat pattern, AT is mainly deposited around the hips, thighs, and lower trunk, leading to a “pear-shaped” shape more often found in premenopausal women, also referred to as peripheral obesity [139,140,141].

Even in the absence of obesity, accumulation of abdominal and central AT, has been related to metabolic and CVD risk in healthy adults in multiple cohort studies [142,143,144]. Additionally, Fu et al. confirmed that higher central fat and central/peripheral fat ratio are significantly associated with increased metabolic risks in adult Chinese women [144]. Furthermore, in children aged 7–13 years old, the central/peripheral ratio was closely positively linked to both insulin resistance and dyslipidemia. This ratio was suggested to be a good predictor for metabolic and CVD risk in normal weight, overweight, and obese children [145].

The association of vAT with metabolic deterioration and cardiometabolic risk, regardless of BMI, has been at the basis of the definition of the MUHNW phenotype [36,37,38,39,40]. Accordingly, multiple studies demonstrated that MUHNW, and also MUO, both associating with poorer metabolic function and inflammatory status, show increased vAT when compared to MHO [3,125,146,147].

Distinct fat compartments within the abdominal AT also lead to different metabolic risk factors. Fox et al. showed that, although both vAT and sAT abdominal compartments are associated with metabolic risk factors, vAT seems to be more strongly correlated with cardiometabolic risk factors [148]. vAT depots show an increased secretion of proinflammatory cytokines (e.g., TNFα) and a reduced secretion of adiponectin, a hormone positively related with whole-body insulin sensitivity. Furthermore, abdominal vAT adipocytes, in comparison to abdominal sAT adipocytes, are more sensitive to catecholamine-induced lipolysis which translates into a greater release of free fatty acids (FFA) into the portal venous system and consequently, to increased lipotoxic effects, mainly in the liver and skeletal muscle. In addition, vAT accumulation was correlated with insulin resistance and hyperinsulinemia. Hence, vAT is nowadays considered as an independent risk factor for T2DM [149,150]. Altogether, the vAT adipocyte profile could explain the deleterious effects observed in vAT accumulation [140,151]. Noteworthy, peripheral fat appears to be concomitant with a lower metabolic risk, which could derive from the less inflammatory nature of the lower-body AT [144,152,153]. As already stated, among the factors underlying the healthier profile found in individuals with MHO are a lower vAT and ectopic fat accumulation (including decreased hepatic steatosis) and more expandable subcutaneous fat depot [13].

Interestingly, and demonstrating how much vAT contributes to determining obesity phenotypes, a novel mathematical model, the visceral adipose index (VAI), was proposed to assess visceral adiposity based on anthropometric and lipid profiles. VAI calculation is assessed by equations comprising waist circumference, BMI, and levels of both low-density lipoprotein (LDL)-cholesterol and triglycerides. Kang et al. showed VAI to be a good predictive tool in determining the conversion of MHO to the more unfavorable MUO phenotype, in both genders [154].

#### Endocrine Disrupting Chemicals: Adipose Tissue Persistent Organic Pollutants and Plastic-Associated Chemicals

Other potential contributors to obesity and metabolic syndrome have been investigated. Among them, are persistent organic pollutants (POPs) that accumulate in AT. These heterogeneous compounds of both natural and anthropogenic origin are highly persistent and present endocrine-disrupting properties [155]. POPs have been studied for their adverse effects on human health. In 2014, Gauthier et al. demonstrated the relationship of POPs with the variation in metabolic risk observed among obese individuals showing that the MHO phenotype is associated with lower plasma levels of POPs as compared with MUO subjects [156]. Although POP accumulation in AT may prevent the systemic effects of these compounds, this tissue can also be a target of their disruptive effects [156]. POP levels, either in sAT and vAT, have been shown to be higher in subjects with evidence of metabolic abnormalities. This pattern was especially evident for vAT, supported by higher vAT POP levels in patients with increased aggregation of metabolic syndrome components and higher 10-year cardiovascular risk based on the Framingham score [157].

Owing to this association, a paradigm shift of the view of POPs as mere obesogen compounds towards their acknowledgement as markers of dysmetabolic obesity was proposed [158]. Indeed, Teixeira et al. showed that the presence of xenoestrogens, such as hexachlorocyclohexane, in vAT and plasma in premenopausal women correlate positively with levels of the proinflammatory chemokine MCP1, thus contributing to a more proinflammatory status [159]. In accordance, it was demonstrated that xenostrogens affect human peripheral blood-derived M1 and M2 macrophage migration, cytokine release, and estrogen-related signaling pathways. These effects were shown to be mediated by either estrogen receptor (ER) α or ERβ and were simultaneous to modulation of NFκB, activator protein-1, JNK, or extracellular signal-regulated kinase signaling pathways [160]. Additionally, Pestana et al. reported exposure to *p,p′*-dichlorodiphenyldichloroethylene (*p,p′*-DDE) in male Wistar rats to be linked to impaired vAT normal function (e.g., decreased tissue development-related genes) and a reduction of the dynamic response to energy surplus, thus translating into exacerbated metabolic syndrome complications [161]. Smink et al. reported an association between prenatal exposure to hexachlorobenzene and increased BMI and weight in children aged 6.5 years in a study involving 482 children [162]. Finally, Lee et al., conducting a 20-year follow-up prospective cohort study with a group of 90 T2DM-free individuals, revealed that *p,p′*-DDE most consistently predicted higher BMI, triglycerides, and HOMA–IR and lower HDL-cholesterol at year 20, when compared to other organochlorine (OC) pesticides. Oxychlordane, *trans*-nonachlor, and hexachlorobenzene could also predict higher triglycerides. Persistent polychlorinated biphenyls with ≥7 chlorides similarly predicted higher BMI, triglycerides, and HOMA-IR and lower HDL-cholesterol at year 20. Accordingly, authors suggested low dose organochlorine pesticides and polychlorinated biphenyls may contribute to obesity, dyslipidemia, and insulin resistance [163]. In the same line, a systematic review and meta-analysis by Cano-Sancho et al. identified prospective associations between exposure to *p,p′*-DDE and increased adiposity from seven epidemiological studies. Two in vivo studies were identified as evidence for positive associations between exposure to *p,p′*-DDT and increased adiposity in rodents. Furthermore, 19 in vivo studies and seven in vitro studies were reported to support the obesogenic effects of the pesticide *p,p′*-DDT and its metabolite *p,p′*-DDE [164]. Very recently, Daniels et al. described 3–9-fold and 9–30-fold higher levels of OC pesticides, respectively, in 120 South Asians of Tamil and Telugu descent, when compared to 120 European Caucasians. Since T2DM is augmented in South Asians and occurs at a lower body weight, blood lipid level, and age in relation to other ethnic populations, one possible explanation for this particular vulnerability is that South Asians have a higher exposure to OC pesticides, according to the same authors [165].

Compared to POPs, plastic-associated chemicals (PACs) such as phthalates and bisphenol A (BPA) are quickly metabolized [166,167,168]. However, these compounds are part of the most produced chemicals worldwide and are present in a very broad range of products such as toys, medical devices, food packaging, personal-care products, and building materials. Due to their ubiquitous nature it is expected that virtually everyone is persistently exposed to these compounds [169,170]. Over the last two decades, multiple studies detected measurable amounts of phthalates, BPA and their metabolites in human samples (e.g., urine, blood, breast milk, amniotic fluid, feces, etc.). Although there is increasing evidence on the involvement of these chemicals in the causation, progression, and susceptibility to metabolic disturbances, it is still a matter of debate and not fully understood [169,170,171,172].

The potential mechanisms of phthalates and BPA action on obesity and glucose metabolism disruption were reviewed by Stojanoska et al. Nuclear receptors seem to be the primary target of these PACs that act as either agonists (complete or partial) or antagonists thus altering regulatory pathways involved in energy homeostasis and metabolism. These nuclear receptors comprise the PPARs α,γ (with a role in adipocyte differentiation and adipogenesis, lipid metabolism, and glucose homeostasis), the estrogen receptors (ERα,β) (with a role in lipid accumulation and adipocyte differentiation), the estrogen-related receptors (ERRγ) (with a role in the regulation of energy homeostasis), the pregnane X receptor (PXR) (with a protective role in the endocrine system by detoxifying against xenobiotics), and the thyroid hormone receptors (TRα,β) (with a role in energy and glucose homeostasis). Besides binding to nuclear receptors, phthalates and BPA exposure was described to increase the orexigenic neuropeptide Y (NPY). Additionally, phthalates were reported to increase reactive oxygen species while BPA associates with the release of inflammatory cytokines IL6 and TNFα [170].

According to cross-sectional analysis of the NHANES 2003–2006 (survey representative of the general adult population of the United States), higher urinary BPA levels were positively associated with the prevalence of T2DM [173] and general and central obesity [174]. Additionally, a study exploring the association between urinary phthalate metabolite concentrations and diabetes among a group of 2350 women who participated in the cross-sectional study NHANES 2001–2008, found that the presence of some phthalates, such as mono-n-butyl phthalate (MnBP), mono-isobutyl phthalate (MiBP), mono-benzyl phthalate (MBzP), mono-(3-carboxy propyl) phthalate (MCPP), and three di-(2-ethylhexyl) phthalate metabolites (∑DEHP), was associated with the prevalence of diabetes [175]. In a cross-sectional study by Hong et al., 296 nondiabetic, reproductive-aged women living in South Korea, were enrolled to explore the relationship between BPA and phthalates levels and insulin resistance and obesity. BPA levels were significantly associated with BMI and waist circumference after adjusting for confounding variables. Consistently, fasting insulin and HOMA-IR were also significantly related to urinary BPA concentration. Authors further reported an increase in BPA levels among metabolically unhealthy women compared to metabolically healthy women. Phthalates were not associated with any of the metabolic parameters [176]. In a pilot study conducted by Piecha et al., the urine levels of bisphenol A and phthalate metabolites were assessed in 168 patients presenting metabolic syndrome with or without, dyslipidemia, hypertension, and T2DM. Four metabolites, mono-n-butyl phthalate (MBP), mono-(2-ethyl-5-hydroxyhexyl) phthalate (MEHHP), mono-(2-ethyl-5-oxohexyl) phthalate (MEOHP), and mono-(2-ethyl-5-carboxypentyl) phthalate (MECPP) had significantly higher levels in diabetic compared to non-diabetic patients. The differences remained significant after adjustment for hypertension, dyslipidemia, age, and BMI [177]. Recently, Shim et al. found a positive association between the concentration of one phthalate metabolite in the urine, MEHHP, and the metabolic syndrome status, involving 5251 participants from the Korean National Environmental Health Survey II (2012–2014), after adjustment for demographic variables. Nevertheless, the authors could not explain the reported association, claiming that chemical properties and health effects of each phthalate metabolite are not well known [178]. Milošević et al. analyzed the presence of 10 different phthalate metabolites in the urine samples of 305 participants of both genders (mono-ethyl phthalate (MEP), mono-(2-ethylhexyl) phthalate (MEHP), mono-methyl phthalate (MMP), mono-n-propyl phthalate (MPP), mono-n-butylphthalate (MBP), mono-iso-allyl phthalate (MiAP), mono-nallylphthalate (MnAP), mono-cyclohexyl phthalate (MCHP), MBzP, and mono-n-octyl phthalate (MOP)). Authors found that exposure to even one phthalate metabolite was associated with an increase in glucose serum levels in all phthalate positive subjects enrolled and divided by three groups: obese, T2DM, and non-obese non-diabetic. Furthermore, they argued phthalate exposure may increase susceptibility for NAFLD development [179]. In a case-control study including 320 children and adolescents (divided into four groups, with or without excess weight and with or without cardiometabolic risk factors), Mansouri et al. found that serum MEHP concentration was associated with higher odds ratio of cardiometabolic risk factors in participants, independently of their weight status. The serum MBP concentration increased the odds ratio of cardiometabolic risk factors only in the normal weight group. Additionally, in young individuals without cardiometabolic risk factors, serum MMP, and MEHHP levels were significantly associated with increased risk of excess weight [171]. Martínez-Ibarra et al. found positive correlations between urinary levels of some phthalates and expression levels of serum miRNAs linked to gestational diabetes mellitus. In particular, MBzP and MEHP levels in urine were associated miR-16-5p and miR-29a-3p expression levels, respectively [180].

Despite the emerging evidence on the role of phthalates and BPA in the development of metabolic syndrome components, a cause-effect relationship between exposure and manifestation of disease remains to be solved as there are still many conflicting results [169,170,172].

Besides all the evidence so far revealing associations between adiposity, TD2M, metabolic disruption, and inflammation, among other effects, further longitudinal and experimental studies are needed to provide firm evidence and confirm whether POPs and PACs are contributors for the metabolic abnormalities observed in MUO or MUHNW phenotypes.

#### Gut Microbiota, Endocannabinoid System, and Inflammasome

The gut microbiota, a diverse microbial community composed of trillions of bacteria, has been recognized as a major environmental factor affecting host metabolic balance and, therefore, is being considered as a virtual endocrine organ [181,182].

Multiple studies comparing obese and lean human subjects revealed significant alterations on gut microbiota composition, notably showing a reduction of Bacteroidetes phylum and a related increase of Firmicutes phylum [183,184,185,186,187]. The reduced Bacteroidetes:Firmicutes ratio has been linked to a more efficient hydrolysis of non-digestible carbohydrates and subsequent increased absorption of calories by the host.

In particular, Turnbaugh et al. identified an increase in Bacteroidetes in individuals losing weight while undergoing a low-calorie diet [185]. Additionally, Jumpertz et al. concluded that nutrient load can affect gut microbiota by showing an association between an augmented energy harvest of ≈150 kilocalories and a 20% increase in Firmicutes associated with a corresponding decrease in Bacteroidetes [188]. Nevertheless, other human studies did not corroborate the association between Bacteroidetes:Firmicutes ratio and obesity [189,190,191,192]. Multiple reports analyzing the microbiota of populations from different regions of the world have started to pinpoint which species, genera, or phyla are altered in individuals with obesity and metabolic disease. Results vary largely between studies and underscore the marked interindividual variations in the gut microbiota composition with genetics, diet, or surrounding environment, for instance [193,194].

Changes in the normal gut microbial composition caused by external factors (e.g., high-fat diet) can lead to dramatic alterations of the symbiotic relationship between the gut microbiota and the host. The microbial imbalance can drive an increase of the intestinal permeability, thus promoting translocation of bacterial products that results in a systemic low-grade elevation in bacterial derived endotoxin lipopolysaccharide (LPS). This condition is termed metabolic endotoxemia [195,196,197]. Increased circulating LPS levels activate the pattern recognition receptor toll-like receptor 4 (TLR4) and trigger proinflammatory and oxidative cascades that contribute to the development of metabolic imbalances observed in obesity and T2DM [196].

For instance, Cani et al. showed that metabolic endotoxemia can dysregulate the inflammatory tone and trigger body weight gain and T2DM in mice [198]. Creely et al. suggested LPS can activate an innate immune response in human AT in T2DM and obesity [199]. In the same line, a population-based cohort study comprising 7169 subjects showed endotoxemia is tightly bound to increased risk of incident T2DM [200]. Finally, a 6-year follow-up study with 2529 middle-aged and older Chinese men and women revealed elevated plasma LPS-binding protein levels correlate significantly with an increased risk of developing metabolic syndrome by triggering the activation of proinflammatory pathways, such as NFκB in macrophages [201]. Noteworthy, *Akkermansia muciniphila*, one of the most abundant single species in the human intestinal microbiota, was reported as a main beneficial bacterium to reduce gut barrier disruption [202,203]. Although the mechanisms of action are not yet fully uncovered, very recently, this effect was attributed to the regulation of tight junction proteins expression by the extracellular lipid bilayer vesicles secreted by *A. muciniphila* [204]. Interestingly, this bacterium is inversely associated with obesity, T2DM, cardiometabolic disease, and low-grade inflammation in humans and there is growing evidence that it may provide protection against certain abnormalities associated with low-grade inflammation [203,205,206]. Noteworthy, very recently a proof-of-concept randomized exploratory study demonstrated, for the first time, that the oral supplementation of *A. muciniphila* (either alive or pasteurized) in obese and overweight volunteer humans for a 3-month period was associated with improvement of several metabolic parameters (e.g., insulin resistance, visceral adiposity, circulating lipids, systemic inflammation markers) [207]. In line, the use of the antidiabetic drug metformin and bariatric surgery have been both correlated with a significant increase in the abundance of *A. muciniphila* [203,208,209].

Obesity and associated inflammatory disorders are related to a dysregulation of the endocannabinoid (eCB) system which, in turn, contributes to an aggravation of the inflammatory tone. Changes in the gut eCB system are involved in the dysregulation of LPS levels and intestinal permeability as well as development of chronic inflammation and dysbiosis of gut microbiota [210,211,212,213]. Noteworthy, the blockade of the cannabinoid receptor type-1 (CB1) was shown to attenuate insulin resistance, glucose intolerance, dyslipidemia, diet-induced obesity, inflammation, and cardiometabolic risk factors [214,215,216].

**Figure 1:**
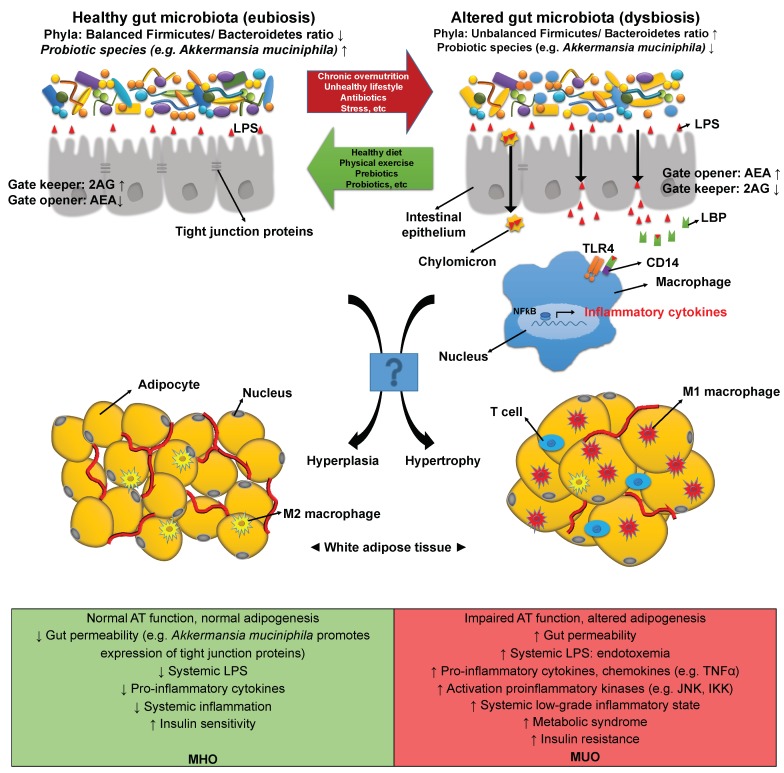
Imbalances in the composition of the gut microbiota can lead to an altered gut-barrier function, and are often observed in several conditions including obesity, related metabolic disorders, and type 2 diabetes. A dysfunctional or “leaky” intestinal tight junction barrier allows augmented translocation of luminal gut-microbiota-derived components, such as LPS, into the blood stream. Increased circulating LPS levels activate the pattern recognition receptor TLR4 and trigger proinflammatory and oxidative cascades that contribute to insulin resistance, macrophage infiltration, secretion of pro-inflammatory cytokines, and lipid accumulation in several organs and tissues, including the AT. The probiotic *Akkermansia muciniphila* preserves the integrity of intestinal barrier function and reduces endotoxemia, possibly by regulating expression of endocannabinoids such as 2AG (augmented), which has been considered as a “gate keeper”, and AEA (decreased), considered a “gate-opener”. During obesity, AT expansion can be mediated by hypertrophy, hyperplasia, or both. MHO individuals are associated with an enhanced adipogenic capacity in comparison to MUO, therefore hyperplasia could potentially be the preferred expansion mechanism of fat tissue in the former individuals. The molecular mechanisms controlling both hyperplasia and hypertrophy have not been fully disclosed so it would be of interest to explore if microbiota and gut-barrier function have any role in regulating the AT expansion during obesity and if this could explain the distinct health profile between MHO and MUO. 2AG, 2-arachidonoylglycerol; AEA, anandamide; AT, adipose tissue; CD14, cluster of differentiation 14); IKK, I kappa B kinase; JNK, c-Jun N-terminal kinase; LBP, lipopolysaccharide binding protein; LPS, lipopolysaccharide; M1, classically activated macrophages; M2, alternatively-activated macrophages; MHO, metabolic healthy obesity; MUO, metabolic unhealthy obesity; NFκB, factor nuclear kappa B; TLR4, Toll-like receptor 4; TNFα, tumor necrosis factor-α.

Recently, Mehrpouya-Bahrami et al. added that the blockade of CB1 in mice attenuates both diet-induced obesity and metabolic disorders and induces alterations in the gut microbiota, namely by increasing the relative abundance of *A. muciniphila* [216]. In the opposite direction, Muccioli et al. showed gut microbiota can modulate endocannabinoid levels in the gut and AT by regulating levels of either cannabinoid receptors agonists such as anandamide (N-arachidonoyl-ethanolamine, AEA) or enzymes needed for their biosynthesis, such as N-acyl-phosphatidylethanolamine-hydrolyzing phospholipase D (NAPE-PLD). Furthermore, authors found that activation of some components of the eCB system in the intestine by an altered gut microbiota increases gut permeability and plasma LPS amount as well as exacerbates gut barrier disruption and peripheral eCB system tone in both the intestinal and ATs. During obesity, the elevated eCB tone and LPS levels are involved in the dysregulation of adipogenesis [217]. Additionally, Liu et al. showed LPS induces a strong production of endogenous ligands of cannabinoid receptors (in particular, AEA) in murine AT macrophages, thus contributing to exacerbation of chronic inflammation in visceral fat, hyperglycemia, and insulin [213]. Importantly, Everard et al. reported that administration of *A. muciniphila* in high-fat diet-fed mice ameliorated the metabolic profile by reversing fat-mass gain, metabolic endotoxemia, AT inflammation, and insulin resistance. *A. muciniphila* administration increases intestinal levels of endocannabinoids that control inflammation (e.g., 2-arachidonoylglycerol (2-AG)) and improves the gut-barrier function [205]. 2-AG was proposed to be an important gate keeper promoting gut barrier function [205]. The associations between dysbiosis, metabolic endotoxemia, and changes in eCB tone as well as inflammasome activation are illustrated in Figure 1.

Apart from the distinction between lean and obese subjects, the gut microbiota is also now identified as a determining factor in the pathogenesis of the MUO phenotype and related comorbidities through increased endotoxemia, intestinal and systemic inflammation, as well as insulin resistance. In accordance, recent studies suggested that a healthy-like gut microbiota profile may contribute to the MHO phenotype [218]. For instance, it remains unanswered whether probiotic bacteria such as *A. muciniphila* could be a factor involved in the differences observed between MHO and MUO.

The multiprotein signaling complex nucleotide-binding oligomerization domain-like receptor (NLRP) 3 inflammasome, which recognizes a wide range of microbial, stress, and damage signals, leads to the activation of caspase-1 and subsequent production of potent proinflammatory cytokines, such as IL1β. Noteworthy, NLRP3 inflammasome components as well as caspase-1 were found to be increased in adipose and liver tissues of obese mice and humans [219]. Vandanmagsar et al. reported a prominent role of NLRP3 inflammasome in inducing obesity and insulin resistance. NLRP3 inflammasome ablated mice do not show obesity-induced inflammasome activation in both fat depots and liver and present better insulin signaling [219].

In spite of the potential role of the NLRP3 inflammasome in metabolic syndrome, activation of this pathway in fat occurs in a late phase of obesity, thus suggesting that NLRP3 inflammasome is actually not a primary etiologic factor in the disease [219,220]. Yet, Esser et al. revealed that MUO phenotype appears to be related to an increased activation of the NLPR3 inflammasome in macrophages infiltrating vAT, and a less favorable inflammatory profile compared with the MHO phenotype [221]. In the opposite direction, NLRP1 inflammasome was described to prevent obesity and metabolic syndrome through IL18 production. Murphy et al. found that mice lacking the NLRP1 inflammasome develop spontaneous obesity due to lipid accumulation. The same effect was observed in mice lacking IL18 [222].

#### Nutrient Intake and Dietary Pattern

In 2017, a large 7-year prospective cohort study using data from 135,335 adults from 18 countries and five continents (Prospective Urban Rural Epidemiology, PURE) concluded that a higher carbohydrate intake was associated with a higher risk of total mortality, in opposition to the intake of total fat and each type of fat (saturated, monounsaturated and polyunsaturated) that were linked to lower risk of total mortality. Additionally, authors reported total fat and saturated and unsaturated fats were not significantly associated with risk of myocardial infarction or CVD mortality [223]. These surprising results are at the basis of a paradigm shift concerning the dietary impact of macronutrients in human disease and mortality. Such conclusions cast doubts on the classical dietary guidelines recommending a substantial reduction of total fat intake, therefore, this subject remains highly controversial [223,224]. Additionally, the majority of studies have been conducted in Western countries (Europe and North America) where nutrition excess is more likely, so it is unsolved whether they are applicable to other populations [223]. Although there is still a major discussion on the impact of each macro- and micronutrient in health, it is well established that the Mediterranean diet is the gold standard of healthy nutrition. Here, the whole is greater than the sum of its parts [225,226]. The Mediterranean diet comprises a balanced combination of fruit, vegetables, fish, cereals, polyunsaturated fats, and a moderate amount of meat and dairy products. Multiple studies have been consensual in associating this diet with decreased morbidity and mortality (mainly from cardiovascular causes). Its health benefits have been attributed to (a) elevated consumption of monounsaturated fat instead of a low fat intake in general; (b) high complex carbohydrate intake, mostly grains and legumes, and (c) high fiber intake, mainly from vegetables and fruit [227]. Micronutrients such as polyphenols and alpha-linolenic acid, may also provide additional cardioprotective effects [227,228]. During a prospective cohort study, the association between Mediterranean diet, metabolic phenotypes, and mortality risk was explored for 1739 adults from the NHANES III survey. Participants were classified as MHO or MUO phenotypes and Mediterranean Diet Scores (MDS) were created to assess the adherence to Mediterranean diet. During a median follow-up of 18.5 years, 12.9% of MHO and 27.1% of MUO subjects died. Noteworthy, a 41% reduction in the risk of all-cause mortality was observed in the MHO phenotype (but not in the MUO phenotype) upon adherence to Mediterranean diet [229]. Very recently, Arenaza et al. examined the adherence to the Mediterranean dietary pattern (MDP) in MHO and MUO phenotypes in European adolescents. In this cross-sectional study comprising 137 overweight/obese adolescents, authors found that adherence to MDP seems to be beneficial to maintaining metabolic health among overweight/obese adolescents [230].

## 4. Conclusions

**Figure 2:**
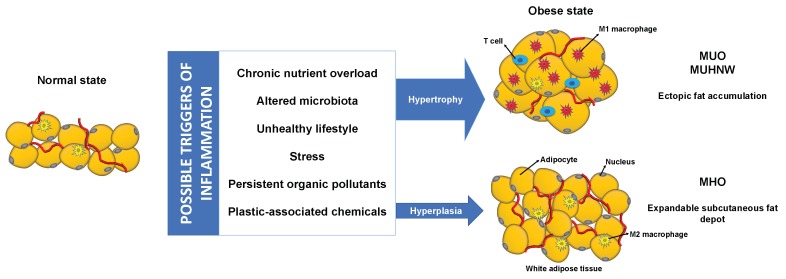
Interplay between factors contributing to inflammatory activation and adipose tissue dysfunction that may underlie the different obesity and metabolic phenotypes. Presence of the depicted possible triggers of inflammation may favor development of hypertrophic dysfunctional adipose tissue, with M1 macrophage recruitment, while impeding adipose tissue growth through hyperplasia with resident M2 macrophages, more prone to metabolic adaptation. M1, classically activated; M2, alternatively activated; MHO, metabolically healthy obese; MUHNW, metabolically unhealthy normal-weight; MUO, metabolically unhealthy obese.

Whatever the trigger for obesity-related inflammation, it is undisputable that it plays a central role in the pathogenesis of obesity comorbidities. Here we report that the inflammatory status associated with adipocyte hypertrophy, proinflammatory AT macrophages, vAT, POPs accumulating in the AT, PACs, and metabolic endotoxemia elicited by an unfavorable gut microbiota along with a subsequent dysregulation of the endocannabinoid tone and the increased activation of the NLPR3 inflammasome may explain why a subset of obese subjects are more prone to develop metabolic disturbances, while others on the opposite side of this spectrum, remain with relatively preserved metabolic function (Figure 2). Meanwhile, the gold standard Mediterranean diet, composed of a diverse group of foods such as fruits, vegetables, whole grains, among others, and with restricted amounts of red meat, sweets, and processed foods, seems to play a major role in preserving the metabolic function. Nevertheless, one cannot ignore that the prognosis for the MHO phenotype remains a matter of intense debate and multiple prospective cohort studies provide evidence that MHO, when compared to their non-obese counterparts, are at higher risk to develop hypertension, T2DM, and metabolic syndrome [38,231]. Still, risk is lower when compared to MUO and MUHNW. Keeping in mind that inflammation may be at the crossroads between obesity phenotypes, interventions aiming to target metabolic inflammation may show promise in counteracting obesity metabolic comorbidities.

## Figures and Tables

**Table 1:** Parameters used in the multiple metabolically healthy obesity definitions.

Parameters Used in the Multiple MHO Definitions	References
CVD diagnosis	[12,55]
Evaluation of insulin sensitivity (determined by euglycemic-hyperinsulinemic clamp, homeostatic model assessment-insulin resistance (HOMA-IR), Matsuda index, insulin suppression test, glucose disposal rate, triglyceride glucose index, and oral glucose tolerance test)	[3,5,6,7,10,11,12,15,16,30,31,32,35,38,40,42,46,48,53,56,59,64,68,71,72,74,77,78,79,84,85,86,87,89]
Determination of systolic and diastolic blood pressure (including information on antihypertensive drug treatment)	[5,6,7,9,10,11,12,16,30,31,32,35,38,42,46,48,52,53,54,55,56,57,58,59,61,64,68,69,70,73,75,76,79,80,81,82,83,84,86,87,88,89]
Circulating lipid profile (apolipoprotein B, triglycerides and total-, low density lipoprotein (LDL)-, and HDL-cholesterol as well as triglycerides/HDL-cholesterol, total-cholesterol/HDL-cholesterol, and % of LDL particles with diameter <255 Å; plus data on associated medication treatment)	[3,5,6,7,9,10,11,12,16,30,31,32,35,38,42,46,48,52,53,54,55,56,57,58,59,61,64,68,69,70,71,73,75,76,77,79,80,81,82,83,84,86,87,88,89]
Circulating glucose levels and related parameters (glycated hemoglobin and history/diagnosis of T2DM as well as use of blood glucose lowering agents/T2DM treatment)	[5,6,7,9,10,11,12,16,30,31,32,35,38,42,46,48,52,53,55,56,57,58,59,61,64,68,69,70,73,75,76,79,80,81,82,83,84,86,87,88,89]
Circulating insulin levels	[12,16,32,54,56,68]
Circulating inflammatory profile (C-reactive protein, fibrinogen and white blood cell count)	[6,11,12,16,31,32,42,52,53,54,56,59,68,75,77,80,83,86,89]
Uric acid levels	[12,16]
Waist circumference	[6,10,11,16,30,31,32,35,52,53,56,61,64,68,69,79,81,82,88,89]
Assessment of cardiorespiratory fitness	[56,89]

**Table 2:** Examples of metabolically healthy obese (MHO) definitions that include inflammatory parameters.

	Ridker et al. 2003 [61]	Song et al. 2007 [75]	Khan et al. 2011 [80]	Hamer et al. 2012a [83]	Hamer et al. 2012b [52]	Iacobellis et al. 2005 [12]	St-Pierre et al. 2005 [54]	Karelis et al. 2008 [77]	Wildman et al. 2008 [86]	Ogorodnikova et al. 2012 [59]
**Glucose**	≥110 mg/dL	Diagnosis of incident T2DM during follow-up	≥100 mg/dL or self-reported use of antidiabetic medications	HbA1c > 6.0% or doctor diagnosed DM	Doctor diagnosed DM	<100 mg/dL or 2-h glucose levels < 140 mg/dL during OGTT	-	-	≥100 mg/dL or antidiabeticmedication use	≥100 mg/dL or DM treatment
**Blood pressure**	SBP/DBP ≥ 135/85 mm Hg	SBP/DBP ≥135/85 mm Hg	SBP/DBP ≥ 130/85 mm Hg or antihypertensive medication use	SBP/DBP > 130/85 mm Hg or hypertension diagnosis or antihypertensive medication use	SBP/DBP > 130/85 mm Hg or hypertension diagnosis or antihypertensive medication use	SBP/DBP < 130/85 mm Hg	SBP/DBP ≥ 135/85 mm Hg	-	SBP/DBP ≥130/85 mm Hg or antihypertensive medication use	SBP/DBP ≥130/85 mm Hg or antihypertensive medication use
**HDL-C**	<50 mg/dL	<50 mg/dL	≤50 mg/dL or lipid lowering medication use	<1.03 mmol/L in men and <1.30 mmol/L in women <40 mg/dL in men and <50 mg/dL in women	<1.03 mmol/L in men and <1.30 mmol/L in women <40 mg/dL in men and <50 mg/dL in women	>40 mg/dL in men and >50 mg/dL in women	<1.0 mmol/L <39 mg/dL	≥1.3 mmol/L ≥50 mg/dL	<40 mg/dL in men, <50 mg/dL in women or lipid-lowering medication use	<40 mg/dL in men, <50 mg/dL in women or lipid-lowering treatment use
**Triglycerides**	≥150 mg/dL	≥150 mg/dL	≥150 mg/dL	≥1.7 mmol/L ≥150 mg/dL	-	<150 mg/dL	≥1.7 mmol/L ≥150 mg/dL	≤1.7 mmol/L ≤150 mg/dL	≥150 mg/dL	≥150 mg/dL
**Other lipid parameters**		-	-	-	-	LDL-C < 130 mg/dL, TC < 200 mg/dL, TG/HDL-C < 3.00 and TC/HDL-C < 4.4	LDL% < 255 Å ≥54.5% ApoB ≥ 1.36 g/L	LDL-C ≤ 2.6 mmol/L LDL-C ≤ 100 mg/dL	-	-
**Insulin sensibility**		-	-	-	-	Insulin < 15 microU/ mL	Insulin ≥ 85.2 pmol/L	HOMA index ≤ 2.7	HOMA-IR > 90th percentile (>5.13)	HOMA-IR > 75th percentile (cut-off = 4.03)
**Inflammatory markers**	(Distribution of CRP levels and stratification for CRP ≥ 3.0 mg/dL vs. ˂3.0 mg/dL)	(Additional stratification for CRP > 3.0 mg/dL vs. ≤3.0 mg/dL)	CRP ≥ 3.0 mg/dL	CRP ≥ 3.0 mg/L	CRP ≥ 3.0 mg/L	WBC < 10,000 cells/mm^3^ and plasma fibrinogen < 4.0 g/L	CRP ≥ 3.0 mg/L	hsCRP ≤ 3.0 mg/L	hsCRP > 90th percentile (>0.1 mg/L)	WBC > 75th percentile (cut-off = 7000 cells/mm^3^)
**Other parameters**	WC ˃ 88 cm	-	-	-	WC > 102 cm in men and >88 cm in women	Uric acid < 5.6 mg/dL in women and <7.0 mg/dL in men; no clinically significant abnormalities on physical examination, no lipid-lowering, hypoglycemic, or antihypertensive drugs, normal thyroid function, no history of metabolic, cardiovascular, respiratory, or other systemic diseases and normal ECG	Nondiabetic individuals free of ischemic heart	-	-	-
**MHO definition**	<3 cardiometabolic abnormalities	<3 cardiometabolic abnormalities	<3 cardiometabolic abnormalities	˂2 cardiometabolic abnormalities	˂2 cardiometabolic abnormalities	All these criteria	<3 cardiometabolic abnormalities	≥4 cardiometabolic abnormalities	˂2 cardiometabolic abnormalities	≤1 cardiometabolic Abnormalities
	Ridker et al. 2003 [61]	Song et al. 2007 [75]	Khan et al. 2011 [80]	Hamer et al. 2012a [83]	Hamer et al. 2012b [52]	Iacobellis et al. 2005 [12]	St-Pierre et al. 2005 [54]	Karelis et al. 2008 [77]	Wildman et al. 2008 [86]	Ogorodnikova et al. 2012 [59]

ApoB, apolipoprotein B; CRP, C-reactive protein; DBP, diastolic blood pressure; DM, diabetes mellitus; ECG, electrocardiogram; HbA1c, glycated hemoglobin; HDL-C, high-density lipoprotein cholesterol; HOMA, homeostasis model assessment; HOMA-IR, homeostasis model assessment of insulin resistance; hsCRP, high-sensitivity C-reactive protein; LDL% < 255 Å, percentage of LDL particles with diameter lower than 255 Å; LDL-C, low-density lipoprotein cholesterol; OGTT, oral glucose tolerance test; SBP, systolic blood pressure; T2DM, type 2 diabetes mellitus; TC, total cholesterol; TG, triglycerides; WBC, white blood cell count; WC, waist circumference. To convert mg/dL to mmol/L, multiply by 0.0259 for HDL-C and LDL-C and by 0.0113 for triglycerides (from Wildman et al. 2008 and http://www.onlineconversion.com/cholesterol.htm).
